# Palmitate Induced IL-6 and MCP-1 Expression in Human Bladder Smooth Muscle Cells Provides a Link between Diabetes and Urinary Tract Infections

**DOI:** 10.1371/journal.pone.0010882

**Published:** 2010-05-28

**Authors:** Andreas Oberbach, Nadine Schlichting, Matthias Blüher, Peter Kovacs, Holger Till, Jens-Uwe Stolzenburg, Jochen Neuhaus

**Affiliations:** 1 Department of Medicine, University of Leipzig, Leipzig, Germany; 2 Faculty of Medicine, Interdisciplinary Centre for Clinical Research, University of Leipzig, Leipzig, Germany; 3 Department of Pediatric Surgery, University of Leipzig, Leipzig, Germany; 4 Department of Urology, University of Leipzig, Leipzig, Germany; Brunel University, United Kingdom

## Abstract

**Background:**

Urinary tract infections (UTI) are more frequent in type-2 diabetes mellitus patients than in subjects with normal glucose metabolism. The mechanisms underlying this higher prevalence of UTI are unknown. However, cytokine levels are altered in diabetic patients and may thus contribute to the development of UTI. Increased levels of free fatty acids (FFA), as observed in obese patients, can induce IL-6 production in various cell types.

Therefore we studied the effects of the free fatty acid palmitate and bacterial lipopolysaccharide (LPS) on interleukin-6 (IL-6) and monocyte chemotactic protein-1 (MCP-1) expression and secretion in cultured human bladder smooth muscle cells (hBSMC).

**Methodology/Principal Findings:**

Biopsies were taken from patients undergoing cystectomy due to bladder cancer. Palmitate or LPS stimulated hBSMC were analysed for the production and secretion of the IL-6, gp80, gp80soluble, gp130, MCP-1, pSTAT3, SOCS3, NF-κB and SHP2 by quantitative PCR, ELISA, Western blotting, and confocal immunofluorescence. In signal transduction inhibition experiments we evaluated the involvement of NF-κB and MEK1 in IL-6 and MCP-1 regulation. Palmitate upregulates IL-6 mRNA expression and secretion via NF-κB dependent pathways in a concentration- and time-dependent manner. MCP-1 was moderately upregulated by palmitate but was strongly upregulated by LPS involving NF-κB and MEK1 dependent pathways. Soluble IL-6 receptor (gp80soluble) was downregulated by palmitate and LPS, while membrane-bound gp80 was moderately upregulated. LPS increased SOCS3 and SHP2, whereas palmitate only induced SOCS3. Secondary finding: most of the IL-6 is secreted.

**Conclusions/Significance:**

Bacterial infection (LPS) or metabolic alterations (palmitate) have distinct effects on IL-6 expression in hBSMC, (i) short term LPS induced autocrine JAK/STAT signaling and (ii) long-term endocrine regulation of IL-6 by palmitate. Induction of IL-6 in human bladder smooth muscle cells by fatty acids may represent a pathogenetic factor underlying the higher frequency and persistence of urinary tract infections in patients with metabolic diseases.

## Introduction

Urinary tract infections (UTI) are more frequent in patients with diabetes mellitus than in subjects with normal glucose metabolism and take a more severe course [1], [2]. Women with diabetes require longer and more aggressive antimicrobial treatment for UTI, and have more recurrences of their UTI than non-diabetic women. The hospitalization due to complications of the UTI occurs significantly more often in women with diabetes [3]. Urine cytokine levels and an increased adherence of the microorganisms to the uroepithelial cells seem to be the main predictors of increased prevalence of both asymptomatic and symptomatic bacteriuria in diabetic patients [4]. Whereas some authors suggest glucosuria as diabetes-specific variable potentially associated with symptomatic infection [5], no association between bacteriuria and indicators of glycemic control, such as the blood glucose level and the glycosylated hemoglobin value, have been found by others [6]. In addition to chronic hyperglycemia, altered fatty acid metabolism belongs to the metabolic alterations associated with type-2 diabetes [7]. However, it is not known whether alterations in circulating free fatty acids (FFA) may contribute to the increased UTI frequency in patients with diabetes.

Bacterial UTI lead to upregulation of cytokines and growth factors and recruitment of inflammatory cells by LPS [8], [9]. IL-6 was shown to be the single most prominent cytokine detected in the urine patients with UTI [10]. Low-grade chronic inflammation is reflected by a 2–3-fold increase in the systemic levels of certain cytokines [11] as well as C-reactive protein (CRP), and an association has been confirmed between low-grade systemic inflammation and type-2 diabetes [12].

There is growing evidence that increased levels of FFA can induce IL-6 production in various cell types, and might therefore be involved in the pathophysiology of UTI [13]–[15]. Elevated FFAs in obese patients may provide a mechanistic link between increased fat mass and the development of insulin resistance, glucose intolerance, and beta-cell dysfunction that promote the onset of diabetes [7], [16]. Palmitate induced accumulation of IL-6 is regulated dependently of c-Jun N-terminal kinase in 3T3-L1 adipocytes [17], but is regulated via NF-κB in myotubes [13], pointing to the existence of potential tissue/cell specific regulatory mechanisms in inflammation. In addition, palmitate could regulate monocyte chemotactic protein-1 (MCP-1) expression in adipose tissue [18], human vascular umbilical vein cells (HUVEC) and rat vascular smooth muscle cells [19].

The aim of the present study was to investigate IL-6 and MCP-1 regulation in hBSMC by acute bacterial infection (LPS stimulation) and metabolic alterations (palmitate) as potential risk factor for chronic bladder inflammation.

## Results

**Figure 1:**
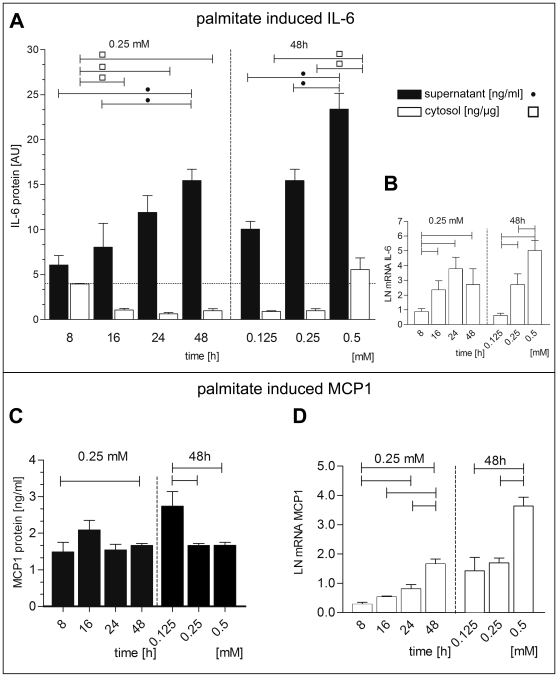
Palmitate effects on IL-6 and MCP-1 mRNA and protein levels. Time- and concentration- dependent regulation of IL-6 and MCP-1 protein (A, C) and mRNA (B, D). IL-6 protein content (A) was measured in cytosol (white bars, ng/µg) and supernatant (black bars, ng/ml), while MCP-1 protein was measured exclusively in supernatant (C, ng/ml). All bars indicate difference to medium control. For each measurement a medium treated control was used. Data are shown as mean and SEM. Significant differences are indicated by lines. mRNA (B, D) was normalized to natural logarithm LN.

Treatment of hBSMC with FFA palmitate (up to 48 hrs) resulted in significantly increased IL-6 protein concentrations in the supernatant, but not in the cytosol (Fig. 1A) A similar pattern was observed for palmitate stimulated IL-6 mRNA expression (Fig. 1B). Increased IL-6 secretion into the medium was dependent on palmitate concentration and time.

**Figure 2:**
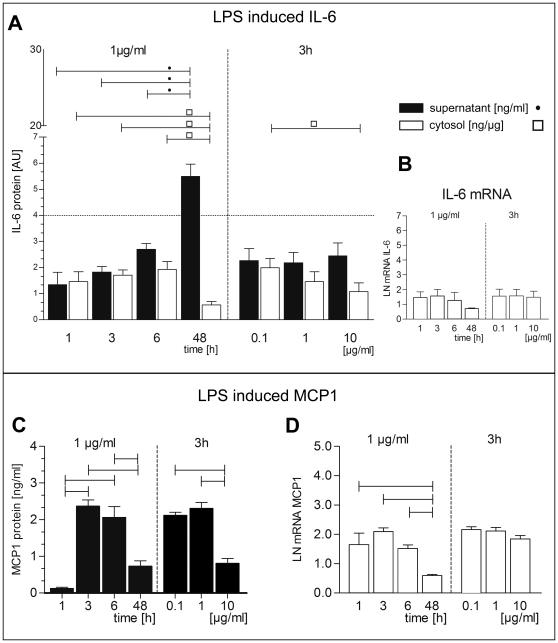
LPS effects on IL-6 and MCP-1 mRNA and protein levels. Time- and concentration- dependent regulation of IL-6 and MCP-1 protein (A, C) and mRNA (B, D). IL-6 protein content (A) was measured in cytosol (white bars, ng/µg) and supernatant (black bars, ng/ml), while MCP-1 protein was measured exclusively in supernatant (C, ng/ml). All bars indicate difference to medium control. For each measurement a medium treated control was used. Data are shown as mean and SEM. Significant differences are indicated by lines. Post-hoc Bonferroni test was used after ANOVA. Significance level was p<0.05. mRNA (B, D) was normalized to natural logarithm LN.

In contrast, stimulation with LPS led to IL-6 protein upregulation in the supernatant while levels decreased with time in the cytosol (Fig. 2A). There was no concentration- dependent regulation of IL-6 mRNA (Fig. 2B). In addition total IL-6 expression was only about 25% of IL-6 protein produced upon palmitate stimulation (Fig. 2A).

While MCP-1 showed similar regulation of the mRNA (Fig. 1D), this concentration- and time-dependent upregulation was not reflected by MCP-1 protein content. Protein expression was constant over 48 hrs and was highest at low palmitate concentration (Fig. 1C). Upon LPS stimulation the MCP-1 protein in the supernatant and in the cytosol decreased time- and concentration-dependent (Fig. 2C). *MCP-1* mRNA showed a comparable downregulation with time but only moderate decrease with increasing concentrations of LPS (Fig. 2D).

**Figure 3:**
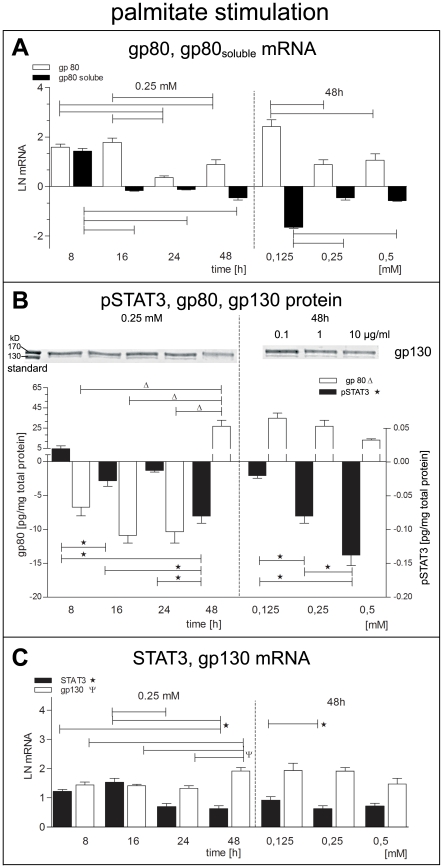
Palmitate effects on the expression of IL-6 receptor subunit gp80, gp80_soluble_, gp130 and pSTAT3. (A) Membrane gp80 receptor mRNA (white bars), soluble gp80 mRNA (black bars). (B) Protein expression of gp80 (white bars) and pSTAT3 (black bars) was measured by ELISA, and membrane gp130 receptor was analysed by Western blot. (C) Gp130 mRNA (white bars), STAT3 mRNA (black bars). Data are shown as mean and SEM. mRNA was normalized to natural logarithm LN.

We found a transient upregulation of the mRNA of IL-6 receptor protein gp80 and its soluble form gp80_ soluble_ (Fig. 3A). After 16 hrs of incubation the gp80 mRNA was still high while the mRNA of gp80_soluble_ was significantly downregulated. This opposite regulatory effect on mRNA levels was most prominent with low palmitate concentration (0.125 mM) but was still present at higher concentrations (Fig. 3A). Gp80 protein was significantly downregulated as early as after 8 hrs incubation lasting up to 24 hrs. However, after 48 hrs incubation with 0.25 mM palmitate the effect was reversed. This high level expression of gp80 was concentration-independent (Fig. 3B).

**Figure 4:**
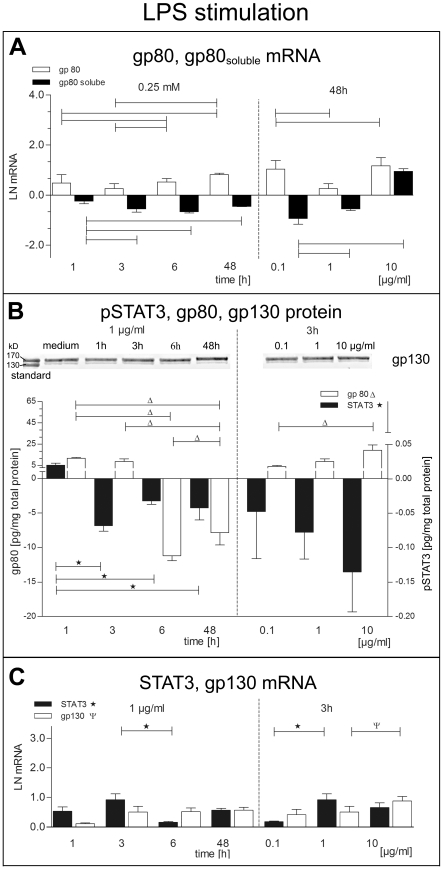
LPS effects on the expression of IL-6 receptor subunit gp80, gp80_soluble_, gp130 and pSTAT3. (A) Membrane gp80 receptor mRNA (white bars), soluble gp80 mRNA (black bars). (B) Protein expression of gp80 (white bars) and pSTAT3 (black bars) was measured by ELISA, and membrane gp130 receptor was analysed by Western blotting. (C) Gp130 mRNA (white bars), STAT3 mRNA (black bars). Data are shown as mean and SEM. mRNA was normalized to natural logarithm LN.

The effects of LPS stimulation on *gp80* and *gp80_soluble_* mRNA are comparable to palmitate induced regulation (Fig. 4A, C), except for a significant initial upregulation of gp80 and gp80_ soluble_ after 8 hrs. After 48 hrs there was an opposite regulation of cytosolic gp80 protein, which was increased by palmitate (0.25 mM) but decreased by LPS (1 µg/ml). Prominent downregulation of pSTAT3 protein was evident in LPS stimulated cells on a short time scale up to 6 hrs (Fig. 4B), whereas there was an upregulation of pSTAT3 protein after 8 hrs of palmitate stimulation (Fig. 3B).

**Figure 5:**
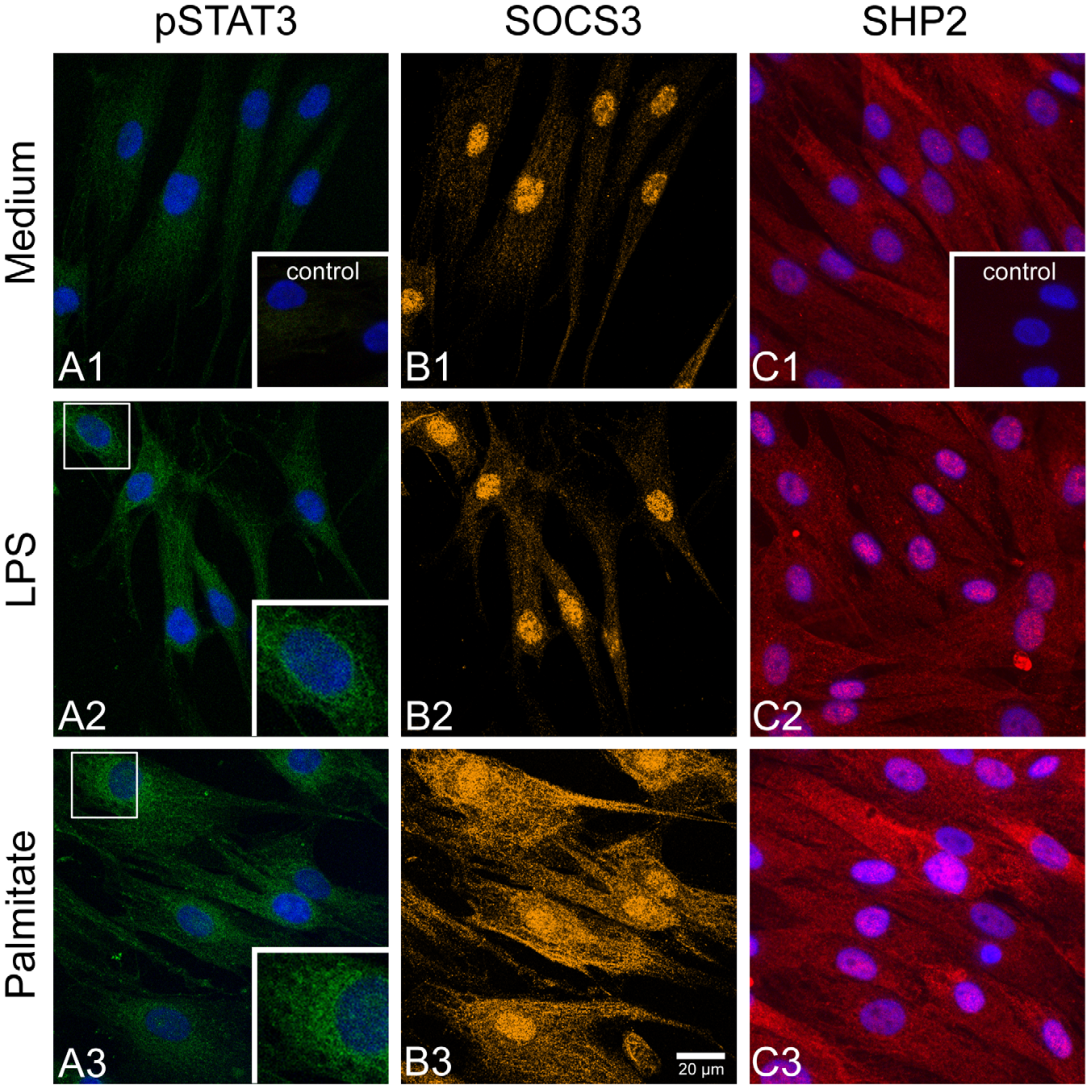
Confocal immunofluorescence of cultures treated for 48 hrs with 1 µg/ml LPS or 0.25 mM palmitate. (A1–C1) Medium control; (A2–C2) LPS treated; (A3–C3) palmitate treated. Cells were double labelled for pSTAT3 (green) and SOCS3 (orange). Monoclonal SHP2 (red) antibody was used in single labelling experiments. Nuclei were stained with DAPI (blue) recorded with standard fluorescence and merged into the confocal images. The scale bar in B3 applies to all images except the insets, which have been enlarged two times.

The membrane IL-6 receptor protein gp130 showed only moderate mRNA upregulation (Fig. 3C). Both, *STAT3* mRNA and protein were significantly downregulated after 24 hrs incubation with 0.25 mM palmitate, which was the most effective concentration at 48 hrs, too (Fig. 3B, C). Confocal imaging revealed perinuclear enrichment of pSTAT3 after 48 hrs stimulation with 1 µg/ml LPS (Fig. 5A2) and even more prominent with 0.25 mM palmitate (Fig. 5A3).

### SOCS3, SHP2

**Figure 6:**
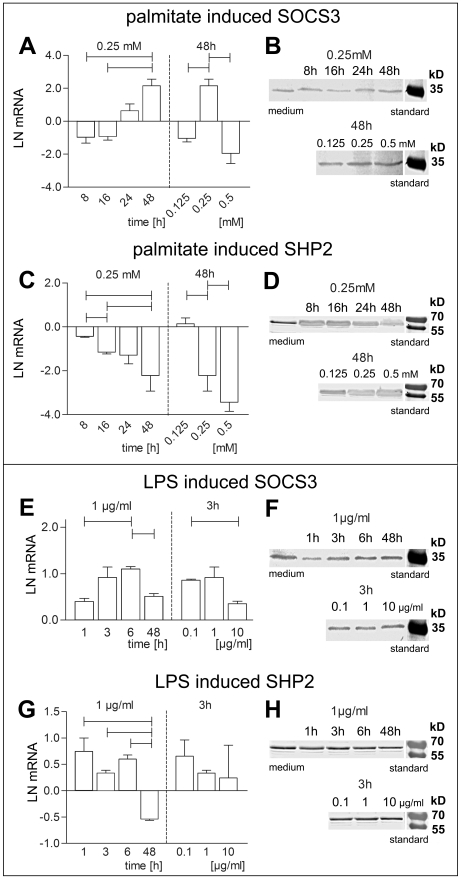
Palmitate and LPS effects on SOCS3 and SHP2. Time- and concentration- dependent palmitate effects on SOCS3 mRNA (A), SHP2 mRNA (C), SOCS3 protein expression (B), and SHP2 protein expression (D). LPS effects on SOCS3 mRNA (E), SHP2 mRNA (F), SOCS3 protein expression (G), and SHP2 protein expression (H). Protein content was analysed in the cytosol using Western blotting analysis. All bars indicate difference to medium control. For each measurement a medium treated control was used. Data are shown as mean and SEM. Significant differences are indicated by lines. mRNA was normalized to natural logarithm LN.

Since SOCS3 and SHP2 are important regulators of IL-6 signaling pathways, we further examined their regulation by palmitate and LPS. *SOCS3* mRNA and SOCS3 protein increased after 48 hrs of palmitate treatment (Fig. 6A, B). *SHP2* mRNA and SHP2 protein levels decreased (Fig. 6C, D). Interestingly, LPS caused upregulation of *SOCS3* mRNA in the first 6 hrs of treatment, but levels returned to baseline level after 48 hrs, while SOCS protein content was highest after 48 hrs (Fig. 6E, F). SHP2 mRNA and protein levels were unaltered after LPS treatment (Fig. 6G, H). These findings were confirmed by confocal immunofluorescence. We found highest immunoreactivity for pSTAT3, SOCS3, and SHP2 after palmitate treatment, while the changes were only moderate in LPS treated cultures (Fig. 5).

### NF-κB p65 regulation and activation

**Figure 7:**
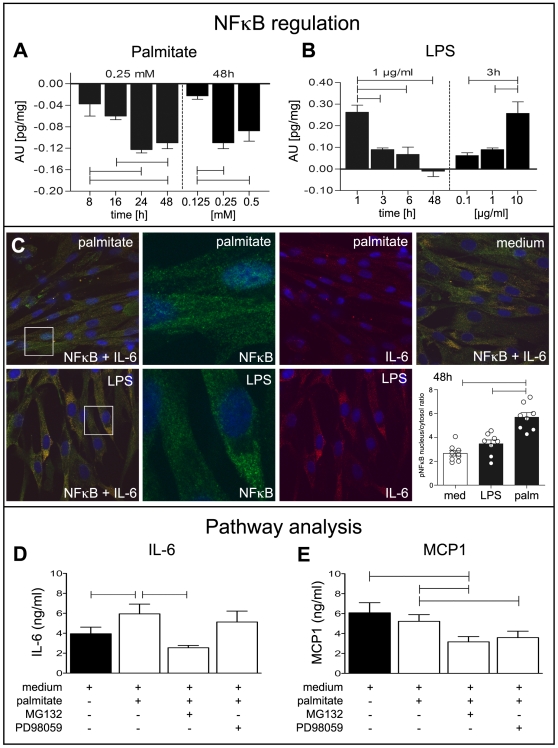
Gene regulation and pathway analysis. Time- and concentration-dependent palmitate and LPS effects on pNF-κB protein expression (A, B). Protein content of pNF-κB was measured in cytosol (A, black bars) and is indicated as difference to medium control (AU in pg/mg) by ELISA. All bars indicate difference to medium control (A, B). For each measurement a medium treated control was used. Confocal double immunofluorescence images demonstrate nuclear translocation of NF-κB (green) and IL-6 (red). Nuclei are depicted by DAPI (blue). Dot blot analysis of phosphorylated NF-κB are shown as ratio of nuclear pNF-κB protein content to cytosolic pNF-κB protein content related to total protein content. Cells were stimulated for 48 h either with 0.25 mM palmitate or with 1 µg/ml LPS (C). Pathway analysis of palmitate induced IL-6 (D) and MCP-1 (E) regulation. Protein content was measured by ELISA in supernatants (ng/ml). Cells were stimulated with palmitate (0.5 mM) and inhibitor of proteasomal degradation of NF-κB (MG132, 40 µM) or MEK1 (PD98059, 20 µM) for 48 hrs. Medium control is indicated as black bar (D, E). Data are shown as mean and SEM. Significant differences are indicated by lines.

NF-κB p65 is the main transcription factor regulating IL-6 gene expression [20]. We found concentration- and time-dependent downregulation of the NF-κB p65 mRNA (Fig. S1A, B) and pNF-κB p65 protein (Fig. 7A, B). This was in striking contrast to the effect of LPS, which led to concentration-dependent upregulation of both, NF-κB p65 mRNA (Fig. S1B) and pNF-κB p65 protein (Fig. 7B).

In double confocal immunofluorecence experiments we found time-dependent translocation of NF-κB p65 into the nuclei as evidence of NF-κB activation after 48 hrs of LPS (1 µg/ml) and palmitate (0.25 mM) stimulation. We found more NF-κB p65 in nuclei of palmitate stimulated cells than after LPS stimulation. To quantify this translocation we performed dot blot analysis using a specific antibody against phosphorylated (Ser536) activated NF-κB p65. Basic ratio was 2.7±0.66 (mean±SD) in medium control and moderate higher in LPS stimulated cells (3.5±0.92), while the ratio was significant higher after 48 h of palmitate stimulation (5.7±1.1).

Pathway analysis by inhibition of NF-κB (MG132) or MEK1 (PD98059) showed that IL-6 was regulated via NF-κB, but not via MEK1, whereas MCP-1 was regulated via NF-κB and MEK1 dependent pathways (Fig. 7D, E).

## Discussion

Here we studied the effects of palmitate and LPS on human bladder smooth muscle cells with the aim to elucidate potential novel mechanisms underlying the observation that urinary tract infections are more frequent in patients with diabetes mellitus and take a more severe course [1], [2]. Defects in the local urinary cytokine secretions and an increased adherence of pathogenic microorganisms to the uroepithelial cells have been discussed as mechanisms for increased prevalence of UTI in patients with diabetes [4]. In a proteom study Yohannes et al. [21] found significant alterations in rat detrusor muscle after streptozotocin-induced diabetes, suggesting that diabetes depending bladder dysfunction may involve dysregulation of structural cell integrity, cell adhesion, proliferation and inflammation [21].

It is well known that during acute infection LPS triggers intracellular signaling cascades via Toll-like receptor TLR4 that rapidly induce inflammatory cytokines that initiate a variety of overlapping immune responses [22].

However, fatty acids and their metabolites act also directly and indirectly to regulate metabolism and immune function through their interactions with specific enzymes [23]. Exaggerated and prolonged postprandial hyperlipidemia is an important characteristic of diabetic dyslipidemia [7], [24].

In the present study we report that palmitate treated human bladder smooth muscle cells (hBSMC) are a source of MCP-1 and IL-6. We used specific inhibitors to interfere with the signaling cascade. We found regulation of IL-6 via NF-κB, which is in line with the regulation of IL-6 by palmitate stimulation in human skeletal muscle cells as reported earlier [13].

Unexpectly, pNF-κB p65 protein content was significantly lower in palmitate stimulated cells as compared to medium control (Fig. 7A). This finding needs further investigation, since on the other hand we found short-term upregulation of pNF-κB p65 by LPS (Fig. 7B). One might suggest that the palmitate stimulus is much stronger than LPS and might therefore lead to increased protein turn-over.

MCP-1 also showed NF-κB dependent regulation as recently reported for IL1β stimulated human aorta smooth muscle cells [25]. However, MCP-1 was downregulated by MEK1 inhibitor (PD98059) in hBSMC, indicating regulation via MEK1 pathway in contrast to IL-6. It is well known that MCP-1 promotes migration of monocytes [26] and this mechanism has also been proposed for bladder inflammation [27]. MCP-1 is also related to insulin resistance and the risk of metabolic syndrome [28].

In hBSMC we found higher secretion of MCP-1 after LPS than after palmitate stimulation. In contrast IL-6 secretion was much higher in palmitate stimulated cells than after LPS treatment (Fig. 1, Fig. 2). To the best knowledge of the authors this is the first study to show that the vast amount of palmitate stimulated IL-6 is secreted. Only basic cytosolic amounts are detectable (Fig. 1). This supports the view that the human bladder smooth muscle cells are involved in immunological processes and may have endocrine function. To examine possible regulatory function of IL-6 onto bladder smooth muscle cells we studied the IL-6 receptor pathway in hBSMC.

The IL-6 signaling cascade depends on the common cytokine receptor family gp130 subunit and the IL-6 receptor specific gp80 subunit forming a hexameric receptor-IL-6 complex. Gp80 exists in a membrane-bound and soluble form, produced as alternative splice variant [29]. For long-term autocrine stimulation by IL-6 the whole receptor complex is needed. We could show, that gp80 mRNA and protein were time-dependent down-regulated by both, palmitate and LSP (Fig. 3, 4). This implies preferential exocrine cytokine signaling by BSMC.

We found short term upregulation of pSTAT3 in LPS and palmitate stimulated cells, but long term downregulation of pSTAT3 (Fig. 3, 4) in conjunction with upregulation of SOCS3 (Fig. 5), which implies negative feedback to IL-6 signaling as reported by Croker et al. [30]. Hence it is evident that both, LSP and palmitate also induced short-term autocrine regulation of IL-6 receptor pathway. However, conditional knockout studies have shown that SOCS3 depletion can induce a number of inflammatory and metabolic disorders [30]. Therefore, upregulation of SOCS3 could be a protection mechanism for the hBSMC to cope with chronically elevated IL-6. At present it is not known which genes are regulated by pSTAT3 in hBSMC.

In conclusion, there is a short-term autocrine IL-6 signaling of BSMC, but a long-term exocrine IL-6 signaling providing cytokine stimulation to local or invading cells equipped with the IL-6 receptor complex.

Our study provides evidence for the urinary bladder smooth muscle cells being an immunologically active tissue, which can be triggered by inflammatory conditions, but which is especially sensitive to metabolic alterations as provoked by the saturated FFA palmitate. In the light of our findings, chronically elevated FFA could well be a risk factor for urinary infections and idiopathic bladder inflammation in diabetes.

## Materials and Methods

### Cell Culture

Human bladder smooth muscle cell (hBSMC) cultures were established from human detrusor muscle after radical cystectomy of tumour patients. Written informed consent was obtained from all patients. The study was approved by the ethics committee of the University of Leipzig (Reg.No. 773). Cells were grown in SMC Growth Medium 2 (PromoCell, Heidelberg, Germany) and subcultured up to the forth passage (P4). At 80% confluence cells were treated with palmitate or with LPS. Palmitate was used in combination with 2% bovine serum albumin (BSA) as carrier of free fatty acids. Medium treated cells were used as control. For pathway analyses, cells were preincubated for 1 h with 20 µM PD98059 (Map Kinase Kinase/Erk Kinase 1 inhibitor) or 40 µM MG123 (proteasome inhibitor; Calbiochem, San Diego, USA). If not indicated otherwise all chemicals were from Sigma-Aldrich, Steinheim, Germany.

### RNA Extraction and Real Time PCR

Total RNA was isolated with RNeasy Mini kit (Qiagen, Hilden, Germany). Quantitative PCR was performed with the ABI 7500 Real Time PCR Instrument (Applied Biosystems, Foster, USA) using the Brilliant SYBR Green QPCR Core Reagent kit (Stratagene, La Jolla, USA) and custom primers (MWG-Biotech, Ebersberg, Germany; Table 1). Human 36B4 (acidic ribosomal phosphoprotein P0) served as internal standard.

**Table 1:** List of primer pairs used for quantitative PCR.

primer	sequence 5′→3′	product length (bp)	binding site
IL-6 forward	GGT ACA TCC TCG ACG GCA TCT	81	exon 2
IL-6 reverse	GTG CCT CTT TGC TGC TTT CAC		exon 3
gp80 forward	GGG GGA AGC ACC ATA ACT TT	232	exon 10
gp80 reverse	ATC TGG GAC TTC AGG CAC AC		exon 10
soluble gp80 forward	CTC CCA GGT TCA AGA AGA CG	183	exon 8/10
soluble gp80 reverse	TGT GGC TCG AGG TAT TGT CA		exon 10
gp130 forward	TGT AGA TGG CGG TGA TGG TA	246	exon 17
gp130 reverse	CCC TCA GTA CCT GGA CCA AA		exon 17
STAT3 forward	TGT GCG TAT GGG AAC ACC TA	170	exon 24
STAT3 reverse	AGA AGG TCG TCT CCC CCT TA		exon 24
SOCS1 forward	CTG GGA TGC CGT GTT ATT TT	245	exon 2
SOCS1 reverse	TAG GAG GTG CGA GTT CAG GT		exon 2
SOCS3 forward	TGG TCA GCT GGT CTC CTT TT	231	exon 2
SOCS3 reverse	CCC ATC CAG GCT GAG TAT GT		exon 2
SH-P2 forward	TGG CCA GAC AGA AGC ACA G	165	exon 3
SH-P2 reverse	GGC TCT GAT CTC CAC TCG TC		exon 3
NF-κB p50 forward	ACA AAT GGG CTA CAC CGA AG	238	exon 23
NF-κB p50 reverse	ATG GGG CAT TTT GTT GAG AG		exon 24
NF-κB p65 forward	CCT GGA GCA GGC TAT CAG TC	213	exon 5
NF-κB p65 reverse	ATC TTG AGC TCG GCA GTG TT		exon 7
MCP1 forward	CCC CAG TCA CCT GCT GTT AT	171	exon 2
MCP1 reverse	TGG AAT CCT GAA CCC ACT TC		exon 3
h36B4 forward	AAC ATG CTC AAC ATC TCC CC	397	exon 6
h36B4 reverse	CCG ACT CCT CCG ACT CTT C		exon 8

### Western Blotting

Cells were washed with PBS and scrapped off in 1× lyses buffer (Cell Signaling, Danvers, USA) supplemented with 1 mM PMSF and 1/100 volume protease inhibitor cocktail (Sigma-Aldrich). After sonication, the cell extracts were cleared by centrifugation. 75 µg of total protein were separated by sodium dodecyl sulfate polyacrylamide (10%) gel electrophoresis and transferred to polyvinylidene fluoride (PVDF) membrane by tank blotting (Mini-PROTEAN II; BioRad, Munich, Germany). Blots were incubated with primary antibodies for 2 h at room temperature or over night at 4°C. Detection and visulization was done with horseradish peroxidase-conjugated anti-rabbit or anti-mouse IgG (1∶1000) for 1 h at room temperature and DAB (FAST 3,3′-diaminobenzidine tablets) as a substrate.

### Dot Blot Analysis

Nuclear and cytosolic extracts were prepared according to Yerneni et al. 1999 [31]. 2 µg total protein of nuclear or cytosolic extracts were transferred in duplicates on nitrocellulose membrane by Dot Blotting (Dot Blot 96 System, Biometra, Goettingen, Germany). After blocking with Odyssee blocking buffer (Licor Biosciences, Bad Homburg, Germany) for 1 h the membranes were incubated with anti-Phospho-NF-κB p65 (Ser536) rabbit IgG (1∶1000; Cell Signaling, Danvers, USA) over night at 4°C. Detection was done with anti-rabbit IRDye 680 (1∶5000; Licor Biosciences) for 2 h. Membranes were scanned with Odyssey Infrared Imager and evaluated by Odyssey Infrared Imaging Software 3.0 (Licor Biosciences). Total protein was visualized by SYPRO Ruby blot stain (BioRad).

### Enzyme-linked Immunosorbent Assay (ELISA)

Intracellular and secreted IL-6 was quantified by Quantikine human IL-6 immunoassay (R&D Systems, Minneapolis, MN). Cell extracts were diluted 1∶10 with calibrator diluent. IL-6 in cell culture supernatants were measured after being centrifuged in 1∶100 diluted samples. IL-6 receptor (gp80) and activated transcription factors (NF-κB, pSTAT3) were determined in undiluted cell extracts by Quantikine human IL-6 sR immunoassay (R&D Systems) and by PathScan Phospho-NF-κB p65 (pNF-κB; Ser536) and PathScan Phospho-STAT3 (pSTAT3; Tyr705) Sandwich ELISA (Cell Signaling). MCP-1 was measured in cell culture supernatants and cell extracts by Quantikine human CCL2/MCP-1 immunoassay (R&D Systems). For quantification the cell culture supernatants were diluted 2 fold and the cell extracts 5 fold with calibration buffer.

### Immunocytochemistry

Cells cultured on cover slips were fixed in 4% buffered paraformaldehyde and incubated overnight at 4°C with primary antibodies (Table 2). Indirect immunofluorescence was performed with secondary antibodies conjugated with Alexa Fluor 488, Alexa Fluor 555 or Alexa Fluor 633 fluorescent dye (1∶500; Invitrogen, Karlsruhe, Germany). Cell nuclei were stained with 4′,6-diamidino-2-phenylindoldihydro-chloride (DAPI). The cells were analysed at a confocal laser scanning microscope LSM-5 Pascal (Carl Zeiss, Jena, Germany).

**Table 2:** List of antibodies used for immunocytochemistry.

antigen	host	type	source	dilution
human IL-6	mouse	monoclonal, IgG_2a_	Acris, Herford, Germany	1∶100
human CD126 (IL-6R)	mouse	monoclonal, IgG_1_	Acris, Herford, Germany	1∶100
human gp130	rabbit	polyclonal, IgG	Santa Cruz Biotechnology, Heidelberg, Germany	1∶100
human pSTAT3 (Tyr705)	mouse	monoclonal, IgG_1_	Cell Signaling Technology, Danvers, USA	1∶100
human SOCS3 (H-103)	rabbit	polyclonal, IgG	Santa Cruz Biotechnology, Heidelberg, Germany	1∶100
human SH-PTP2 (C-18)	rabbit	polyclonal, IgG	Santa Cruz Biotechnology, Heidelberg, Germany	1∶100
human NF-κB p65 (F-6)	mouse	monoclonal, IgG_1_	Santa Cruz Biotechnology, Heidelberg, Germany	1∶100

### Statistical Analysis

Complete data analysis was performed using Prism 5.0 (GraphPad Software, La Jolla, USA) statistical software. The data are presented as the mean +/− SEM from at least three independent experiments. Statistical differences were analyzed by ANOVA. A p-value <0.05 was considered statistically significant.

## Supporting Information

Figure S1 Gene regulation of NF-κB p65. Time- and concentration-dependent palmitate and LPS effects on NF-κB p65 mRNA expression (A, B). All bars indicate difference to medium control. For each measurement a medium treated control was used. Data are shown as mean and SEM. Significant differences are indicated by lines. mRNA was normalized to natural logarithm LN.
(0.30 MB TIF)

## References

[1] Benfield T, Jensen JS, Nordestgaard BG (2007). Influence of diabetes and hyperglycaemia on infectious disease hospitalisation and outcome.. Diabetologia.

[2] Stapleton A (2002). Urinary tract infections in patients with diabetes.. Am J Med.

[3] Schneeberger C, Stolk RP, Devries JH, Schneeberger PM, Herings RM (2008). Differences in the pattern of antibiotic prescription profile and recurrence rate for possible urinary tract infections in women with and without diabetes.. Diabetes Care.

[4] Hoepelman AI, Meiland R, Geerlings SE (2003). Pathogenesis and management of bacterial urinary tract infections in adult patients with diabetes mellitus.. Int J Antimicrob Agents.

[5] Harding GK, Zhanel GG, Nicolle LE, Cheang M (2002). Antimicrobial treatment in diabetic women with asymptomatic bacteriuria.. N Engl J Med.

[6] Geerlings SE, Stolk RP, Camps MJ, Netten PM, Collet TJ (2000). Risk factors for symptomatic urinary tract infection in women with diabetes.. Diabetes Care.

[7] Wilding JP (2007). The importance of free fatty acids in the development of Type 2 diabetes.. Diabet Med.

[8] Bouchelouche K, Alvarez S, Horn T, Nordling J, Bouchelouche P (2006). Human detrusor smooth muscle cells release interleukin-6, interleukin-8, and RANTES in response to proinflammatory cytokines interleukin-1beta and tumor necrosis factor-alpha.. Urology.

[9] Saban MR, Hellmich H, Nguyen NB, Winston J, Hammond TG (2001). Time course of LPS-induced gene expression in a mouse model of genitourinary inflammation.. Physiol Genomics.

[10] Otto G, Braconier J, Andreasson A, Svanborg C (1999). Interleukin-6 and disease severity in patients with bacteremic and nonbacteremic febrile urinary tract infection.. J Infect Dis.

[11] Ross R (1999). Atherosclerosis—an inflammatory disease.. N Engl J Med.

[12] Festa A, D'Agostino RJ, Tracy RP, Haffner SM (2002). C-reactive protein is more strongly related to post-glucose load glucose than to fasting glucose in non-diabetic subjects; the Insulin Resistance Atherosclerosis Study.. Diabet Med.

[13] Weigert C, Brodbeck K, Staiger H, Kausch C, Machicao F (2004). Palmitate, but not unsaturated fatty acids, induces the expression of interleukin-6 in human myotubes through proteasome-dependent activation of nuclear factor-kappaB.. J Biol Chem.

[14] Staiger H, Staiger K, Stefan N, Wahl HG, Machicao F (2004). Palmitate-induced interleukin-6 expression in human coronary artery endothelial cells.. Diabetes.

[15] Chavez JA, Summers SA (2003). Characterizing the effects of saturated fatty acids on insulin signaling and ceramide and diacylglycerol accumulation in 3T3-L1 adipocytes and C2C12 myotubes.. Arch Biochem Biophys.

[16] Eitel K, Staiger H, Rieger J, Mischak H, Brandhorst H (2003). Protein kinase C delta activation and translocation to the nucleus are required for fatty acid-induced apoptosis of insulin-secreting cells.. Diabetes.

[17] Davis JE, Gabler NK, Walker-Daniels J, Spurlock ME (2009). The c-Jun N-terminal kinase mediates the induction of oxidative stress and insulin resistance by palmitate and toll-like receptor 2 and 4 ligands in 3T3-L1 adipocytes.. Horm Metab Res.

[18] Chakrabarti SK, Cole BK, Wen Y, Keller SR, Nadler JL (2009). 12/15-lipoxygenase products induce inflammation and impair insulin signaling in 3T3-L1 adipocytes.. Obesity (Silver Spring).

[19] Ishikado A, Nishio Y, Yamane K, Mukose A, Morino K (2009). Soy phosphatidylcholine inhibited TLR4-mediated MCP-1 expression in vascular cells.. Atherosclerosis.

[20] Ajuwon KM, Spurlock ME (2005). Palmitate activates the NF-kappaB transcription factor and induces IL-6 and TNFalpha expression in 3T3-L1 adipocytes.. J Nutr.

[21] Yohannes E, Chang J, Christ GJ, Davies KP, Chance MR (2008). Proteomics analysis identifies molecular targets related to diabetes mellitus-associated bladder dysfunction.. Mol Cell Proteomics.

[22] Akira S, Uematsu S, Takeuchi O (2006). Pathogen recognition and innate immunity.. Cell.

[23] Zhao S, Yang YN, Song JG (2004). Ceramide induces caspase-dependent and -independent apoptosis in A-431 cells.. J Cell Physiol.

[24] Stampfer MJ, Rimm EB (1996). Folate and cardiovascular disease. Why we need a trial now.. JAMA.

[25] Lim JH, Um HJ, Park JW, Lee IK, Kwon TK (2009). Interleukin-1beta promotes the expression of monocyte chemoattractant protein-1 in human aorta smooth muscle cells via multiple signaling pathways.. Exp Mol Med.

[26] Reape TJ, Groot PH (1999). Chemokines and atherosclerosis.. Atherosclerosis.

[27] Bouchelouche K, Andresen L, Alvarez S, Nordling J, Nielsen OH (2006). Interleukin-4 and 13 Induce the Expression and Release of Monocyte Chemoattractant Protein 1, Interleukin-6 and Stem Cell Factor From Human Detrusor Smooth Muscle Cells: Synergy With Interleukin-1beta and Tumor Necrosis Factor-alpha.. J Urol.

[28] Kanda H, Tateya S, Tamori Y, Kotani K, Hiasa K (2006). MCP-1 contributes to macrophage infiltration into adipose tissue, insulin resistance, and hepatic steatosis in obesity.. J Clin Invest.

[29] Rose-John S, Scheller J, Elson G, Jones SA (2006). Interleukin-6 biology is coordinated by membrane-bound and soluble receptors: role in inflammation and cancer.. J Leukoc Biol.

[30] Croker BA, Krebs DL, Zhang JG, Wormald S, Willson TA (2003). SOCS3 negatively regulates IL-6 signaling in vivo.. Nat Immunol.

[31] Yerneni KK, Bai W, Khan BV, Medford RM, Natarajan R (1999). Hyperglycemia-induced activation of nuclear transcription factor kappaB in vascular smooth muscle cells.. Diabetes.

