# Supplementary figures and images for: Palmitate Induced IL-6 and MCP-1 Expression in Human Bladder Smooth Muscle Cells Provides a Link between Diabetes and Urinary Tract Infections
Source: PLoS One. 2010 May 28;5(5):e10882. doi: 10.1371/journal.pone.0010882 (PMC2878332; doi:10.1371/journal.pone.0010882)

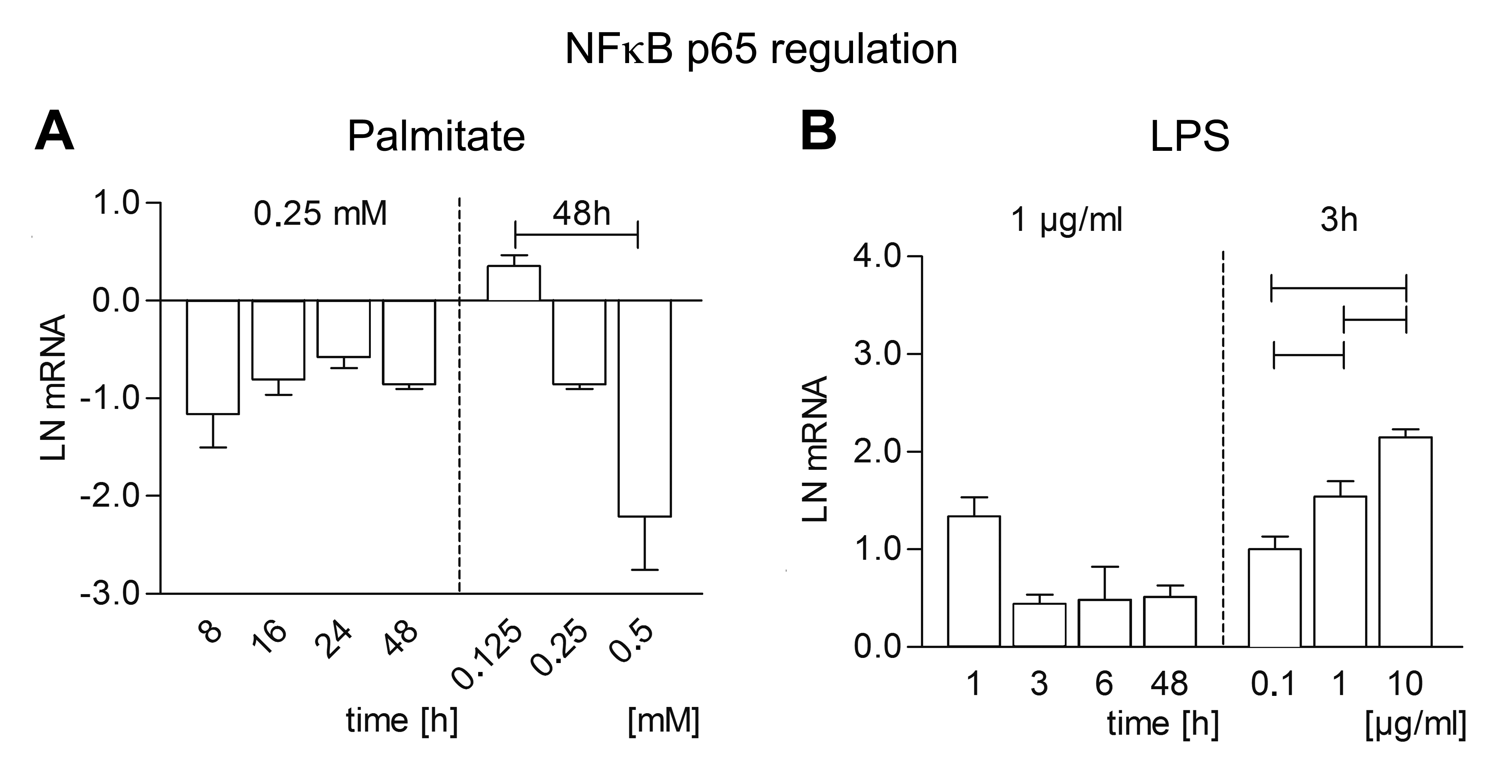

Supplement: Figure S1 — Gene regulation of NF-κB p65. Time- and concentration-dependent palmitate and LPS effects on NF-κB p65 mRNA expression (A, B). All bars indicate difference to medium control. For each measurement a medium treated control was used. Data are shown as mean and SEM. Significant differences are indicated by lines. mRNA was normalized to natural logarithm LN. (0.30 MB TIF) [file pone.0010882.s001.tif]
